# Energy-Efficient Wearable EPTS Device Using On-Device DCNN Processing for Football Activity Classification

**DOI:** 10.3390/s20216004

**Published:** 2020-10-22

**Authors:** Hyunsung Kim, Jaehee Kim, Young-Seok Kim, Mijung Kim, Youngjoo Lee

**Affiliations:** 1 Department of Electrical Engineering, Pohang University of Science and Technology (POSTECH), 77 Cheongam-ro, Pohang 37673, Korea; hyunsungkim@postech.ac.kr (H.K.); jayjay@postech.ac.kr (J.K.); 2 Institute of Artificial Intelligence, Pohang University of Science and Technology (POSTECH), 77 Cheongam-ro, Pohang 37673, Korea; andyyskim@postech.ac.kr; 3 Sports AIX Graduate Program, Pohang University of Science and Technology (POSTECH), 77 Cheongam-ro, Pohang 37673, Korea; mijungkim@postech.ac.kr

**Keywords:** electronic performance and tracking system, sports wearable device, energy-efficient sensor control, on-device DCNN processing

## Abstract

This paper presents an energy-optimized electronic performance tracking system (EPTS) device for analyzing the athletic movements of football players. We first develop a tiny battery-operated wearable device that can be attached to the backside of field players. In order to analyze the strategic performance, the proposed wearable EPTS device utilizes the GNSS-based positioning solution, the IMU-based movement sensing system, and the real-time data acquisition protocol. As the life-time of the EPTS device is in general limited due to the energy-hungry GNSS sensing operations, for the energy-efficient solution extending the operating time, in this work, we newly develop the advanced optimization methods that can reduce the number of GNSS accesses without degrading the data quality. The proposed method basically identifies football activities during the match time, and the sampling rate of the GNSS module is dynamically relaxed when the player performs static movements. A novel deep convolution neural network (DCNN) is newly developed to provide the accurate classification of human activities, and various compression techniques are applied to reduce the model size of the DCNN algorithm, allowing the on-device DCNN processing even at the memory-limited EPTS device. Experimental results show that the proposed DCNN-assisted sensing control can reduce the active power by 28%, consequently extending the life-time of the EPTS device more than 1.3 times.

## 1. Introduction

**Figure 1:**
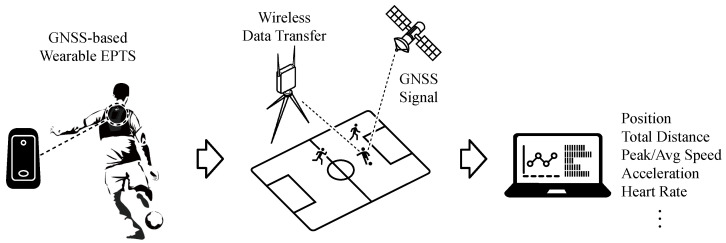
A wearable electrical performance tracking system (EPTS) in football matches.

Since the German national football (The term football in this work refers to soccer or association football.) team dominated the World Cup in 2014 with various IT technologies [1], the use of an electronic performance tracking system (EPTS) has been gaining huge popularity in the football industry and now has been standardized by FIFA to be used even at the international matches [2]. Basically, an EPTS device can be attached to the body of football players during the match (or training) time, sensing a number of data related to the athletic and strategic performances. As exemplified in Figure 1, the EPTS device may have a positioning system assisted by numerous calibration sensors [3,4,5], collecting quantitative data such as total distance covered, peak/average speed, or other physiological data. These on-site measurements can be used for analyzing the physical workloads of each player, providing valuable insights to optimize the performance [6,7]. Considering the agile movements of football players, the accurate positioning solution is one of the key technologies, which can be realized by an optical-based tracking system (OTS), a local positioning system (LPS), or a global navigation satellite system (GNSS) [8]. While the OTS and LPS show a high sampling rate and measurement accuracy, both systems typically require high-cost calibration infrastructures installed around the stadium [8]. On the other hand, the GNSS-based EPTS device is usable in any open field, whereas the position and speed are directly measured using satellite signals, as shown in Figure 1. Due to the intrinsic errors of the GNSS module [9,10], the MEMS-based inertia measurement unit (IMU) module is in general integrated into the GNSS-based EPTS device to provide more accurate information [11,12,13].

Even though the calibration schemes are essentially applied for minimizing the existing measurement errors, many researches have revealed that the accessing rate of GNSS dominantly determines the measurement accuracy of wearable EPTS devices [7,14,15]. Therefore, the current wearable EPTS devices frequently perform the GNSS sampling operations to retain the validity and the reliability of measurements. For example, the state-of-the-art wearable EPTS device, which delivers comparable accuracy to the LPS (or OTS) solutions, includes a 10 Hz GNSS receiver for the main localizing operation and also utilizes the IMU module running at a speed of 100 Hz for the proprietary filter algorithm [15,16]. As activating the GNSS module, as well as other assisting sensors consumes a huge amount of energy, the contemporary approaches typically suffer from the battery overheads to support the required operating time, increasing the device form factor accordingly. Note that the related standard clearly indicates the minimum safety requirement of wearable EPTS devices [17], and it is widely reported that the small form factor of the EPTS device is essential for preventing unwanted injuries [18]. Despite the quantitative feedback to players, as a result, many players still disapprove of using this technology at the important matches due to the uncomfortable experience. Hence, improving the energy efficiency of wearable EPTS devices without degrading the accuracy performance is still an open problem to reduce the required battery size and will play an important role in overcoming the limitations of current approaches in real environments.

In this paper, we propose a novel approach to reduce the overall energy consumption of the wearable EPTS device. To realize the experimental environments, we first design a prototype of the GNSS-based EPTS device, and then, the adaptive GNSS control method is newly introduced by categorizing the football movement types of players using a deep convolutional neural network (DCNN). Considering the tight memory size of the EPTS device, the previous DCNN model for recognizing the human activities is compressed to remove as many of the redundant parameters as possible, allowing the on-device DCNN processing by consuming negligible energy at the embedded processor. Experimental results show that the proposed idea significantly reduces the number of GNSS accesses while supporting similar distance and speed measurement errors compared to the baseline operations, consequently saving the overall energy consumption of the EPTS device by 28%.

The rest of this paper is organized as follows. Section 2 presents the detailed architecture of the prototype EPTS device, and the proposed energy-aware sensing-rate control method is introduced in Section 3. The experimental results under the field-level tests are shown and compared to the other works in Section 4. Finally, concluding remarks are made in Section 5.

## 2. Proposed Wearable EPTS Device

### 2.1. System Architecture

**Figure 2:**
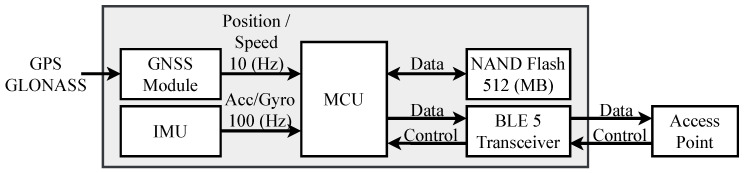
A block diagram of the proposed wearable EPTS device.

Figure 2 illustrates the block diagram of the proposed wearable EPTS device prototype that includes a GNSS module [19], an IMU module [20], a micro-controller unit (MCU) [21], NAND flash memory, and wireless communication modules using BLE5protocols, which is generally adapted by commercial EPTS devices [22,23,24]. For the baseline EPTS operation, the accessing frequency of GNSS module was set to 10Hz, measuring the position, as well as the speed of players every 100 ms by receiving the global positioning system (GPS) signal and the global navigation satellite system (GLONASS) signal concurrently. The nine axis MEMS-based IMU device consists of an accelerometer, a gyroscope, and a magnetometer, where the first two internal sensors generate the measurement data every 10 ms. The magnetometer in the IMU module is reserved for the future extension, and thus, we disabled the corresponding parts for low-energy operations. From the IMU measurements, the firmware program basically computes the total amount of acceleration by performing the attitude estimation filter [25]. Then, the linear Kalman filter is applied to compensate for the intrinsic speed errors by combining the measured speed from the GNSS unit with the estimated net acceleration [26]. Note that the sampling time of sensor measurements are synchronized at the firmware level to provide accurate filtering operations. In addition to the sensor-related EPTS operations, in the proposed EPTS device, the BLE5-based radio transceiver was adopted to realize the stable real-time tracking and the efficient remote control of the EPTS device with low energy consumption. When the EPTS cannot use wireless communication, the sensing data are temporally stored in the NAND flash memory of 512 MB, which is enough to keep all the data for the match time. To manage the overall EPTS operations, we also implemented the control firmware on the ARM Cortex-M4 MCU, which disables the inactivated components to reduce the power consumption in standby mode.

### 2.2. Evaluation of Baseline EPTS operations

**Figure 3:**
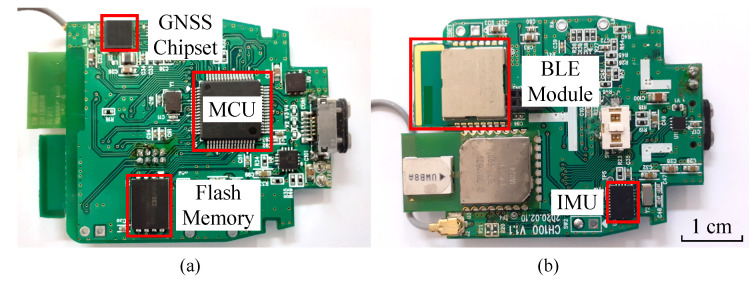
A circuit board of the proposed wearable EPTS device: (**a**) front-side and (**b**) back-side.

Based on the system architecture shown in Figure 2, we realized a prototype EPTS device having a form factor of 53mm×32mm×9mm, where the top and bottom views of the prototype are shown in Figure 3. As discussed in the prior subsection, the basic EPTS operation sets the GNSS and the IMU sensing rates to be 10 Hz and 100 Hz, respectively, whereas the embedded MCU operates at a speed of 80 MHz for performing the overall firmware programs managing sensors and data flows. Compared to our original EPTS solution described in [27], note that the proposed version reduces the device size by 40% through the compact design of the circuit board while providing measurements at the same sensing rates. The BLE5 unit continuously transfers the position, speed, and IMU measurements at a speed of 125 Kbps. Using the coin-sized 550 mAh battery, the baseline operation on the prototype EPTS device lasts about five hours, which is comparable to the commercialized products having similar form factors [22,23,24].

**Figure 4:**
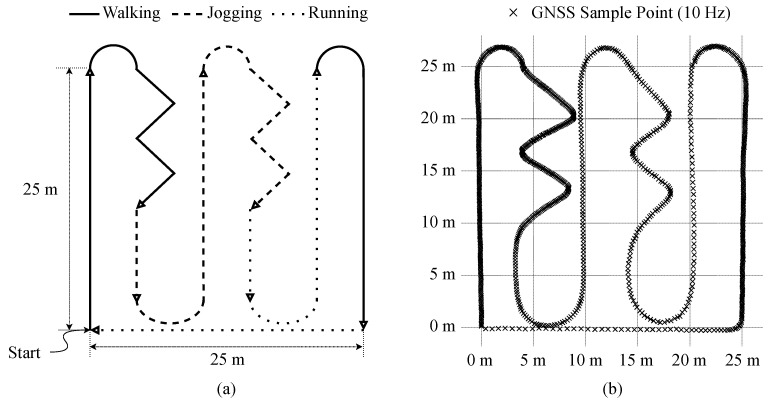
(**a**) Standardized testing scenario. (**b**) The measured trajectory using the proposed device.

In order to evaluate the accuracy of the baseline EPTS operations, we performed the standardized testing scenario from [28]. As depicted in Figure 4a, more precisely, this trajectory testing includes different movements that occurred in football matches such as walking, running, and turning. For the given trajectory, Figure 4b shows the sampling positions from the prototype EPTS device by activating the GNSS module every 100 ms. The quality of the acquired data from the EPTS device was investigated by comparing with the ground truth using two metrics. To analyze the quality of the acquired information from the EPTS device, it is necessary to prepare the ground truth information of the location and speed at each sampling point, and then, two error metrics $E_{d}$ and $E_{s}$ are investigated to represent the errors of distance and speed, respectively [15,28,29]. For the sake of simplicity, we define $d_{E}$ and $s_{E}$ to denote the measured information of the distance between two adjacent samples and the speed value at each sample, respectively. Then, the error metrics $E_{d}$ and $E_{s}$ are calculated as follows:

(1) $$E_{d}=\sqrt{\frac{1}{n}\sum_{i=1}^{n}{\left(d_{E}^{\left(i\right)}-d_{G}^{\left(i\right)}\right)}^{2}},\;E_{s}=\sqrt{\frac{1}{n}\sum_{i=1}^{n}{\left(s_{E}^{\left(i\right)}-s_{G}^{\left(i\right)}\right)}^{2}},$$

where *n* is the total number of samples and $d_{G}$ and $s_{G}$ indicate the distance and the speed of the pre-defined ground truth, respectively. Similarly, $d_{E}$ and $s_{E}$ represent the measured data of the distance and speed from the EPTS device, respectively. As depicted in Figure 4, note that our prototype EPTS device supports quite accurate measurements of athletic movements, i.e., $E_{d}=0.076\;m$ and $E_{s}=0.47\;m\cdot s^{-1}$, which are comparable to the contemporary EPTS products targeting national-level football matches [22,23,24].

As the energy consumption of the EPTS device is directly related to the operating time, as well as the form factor, it is necessary to deeply understand the power consumption of each building block. For the quantitative analysis, we evaluated the energy consumption of the prototype EPTS device performing the baseline operations. Table 1 summarizes how the three major components dissipate the power, where the effects of other modules are negligible in terms of power consumption. It is clear that the GNSS module is the most power-hungry unit due to the frequent activation of RFIC circuits followed by the antenna component, consuming more than half of the total power budget. In order to develop the energy-efficient EPTS device, therefore, it is necessary to develop the advanced control scheme of GNSS operations, which reduces the number of GNSS samples without degrading the error metrics, i.e., $E_{d}$ and $E_{s}$.

## 3. Proposed Power Optimization Method

### 3.1. Activity-Aware GNSS Control

**Figure 5:**
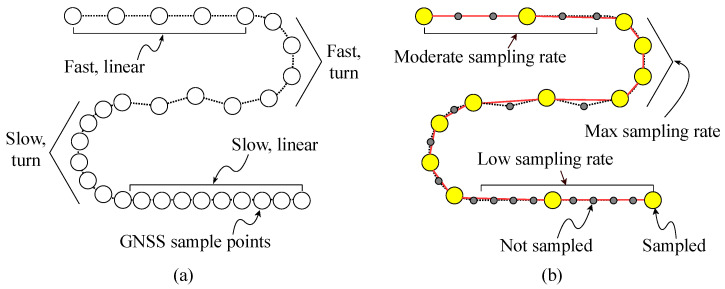
(**a**) A trajectory recovered by the baseline sampling associated with the fully-activated GNSS module and (**b**) a trajectory recovered by the activity-aware sampling with the reduced number of GNSS accesses.

It is well known that the energy consumption of sensor-based mobile devices can be remarkably saved by introducing the adaptive sensor control strategy associated with domain-specific knowledge [30,31]. For example, Krause et al. [32] investigated the trade-off of adaptive sensor control strategies between the human-motion classification performance and the sampling rate, which can be specialized to handle the support vector machine (SVM) and Markov chain model. Andersson et al. [33] introduced the two step control method for activating the power-hungry sensors based on the sensing results of low-power but less-accurate sensors. Considering the movements of football players as described for the tracking test scenario in [28], inspired by the prior works, we designed an advanced firmware-level optimization to reduce the activation frequency of the GNSS module depending on the athletic actions of players. By categorizing the movement types using the current sensing data, as conceptually illustrated in Figure 5, more precisely, we can disable the GNSS receiver for a moment when the player stays from a certain position during the match time. For the case study, in this work, we define six activities that frequently occur at football matches, as summarized in Table 2. To minimize the measurement errors caused by the reduced number of sensing samples, therefore, it is important to develop a simple but accurate algorithm on the embedded MCU for categorizing these pre-defined football activities.

For the straight-forward classification, similar to the prior work from [34], we may directly use the speed measurements from the GNSS unit to set the intuitive thresholds to classify some human activities. For example, the athlete’s movement can be determined based on the fixed speed criteria defined in related papers [29,35], and then, the GNSS sampling rate for each activity can be adjusted to eliminate over-sampling data, as described in Table 2. However, the speed value can only provide hints for categorizing the linear movements, and this approach cannot capture the rotation-related motions. Therefore, the energy-reduction from the straight-forward recognition is marginal due to the limited capability for categorizing the movement types of football players. In order to provide precise control of sensor modules, by using additional sensing signals from the IMU module, we develop the DCNN-based recognition of football activities, further reducing the energy consumption of the EPTS device.

### 3.2. Proposed DCNN-Based Classification of Football Activities

Recently, like the other classification issues [36,37,38,39], the algorithm-level performance of the human activity recognition (HAR) problem from IMU data has been remarkably improved by accepting DCNN approaches [40,41,42,43]. For example, the 1D CNN from [44] utilizes the sensing data of a three axis accelerometer, finding the temporal features along each channel. Extending the dimension of the CNN architecture can capture the correlated features in multiple channel domains, improving the recognition accuracy of the HAR problem [41]. Applying the pre-processing for multiple sensor measurements, furthermore, the quality of the CNN-based HAR system can be further improved as reported in [42,43]. For open-source datasets of motions in daily life [40,45,46,47], Table 3 summarizes the performance of different CNN-based HAR systems in terms of the recognition accuracy, as well as the required memory size for storing the trained network. Note that the recent work from [43] offers the smallest memory footprint among the existing works. Hence, we design a compact DCNN model based on the work from [43], which is dedicated to recognizing the football activities at the resource-limited device.

**Figure 6:**
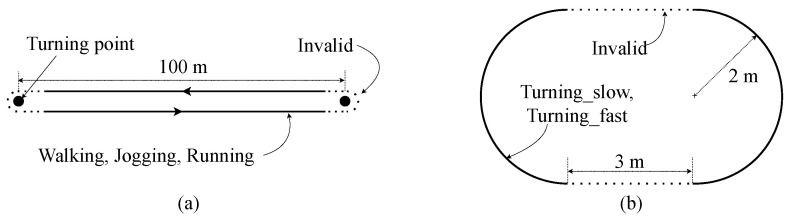
Circuit diagrams for collecting samples of (**a**) linear movements and (**b**) turning movements.

Instead of using the open-source dataset directly [40,45,46,47], in order to generate a compact DCNN design, we collected actual data samples using the prototype EPTS devices. The custom dataset contains total 28,712 one second long samples of IMU measurements from five male subjects (age = 24.6 ± 2.2 years; height = 175.8 ± 3.7 cm; weight = 68.6 ± 3.6 kg). For the balanced data acquisition, in addition to using the standardized trajectory shown in Figure 4a, we used two more tracks for collecting samples, which are more focused on the straight and rotation activities, as illustrated in Figure 6a and Figure 6b, respectively. Note that the acquired IMU samples were divided into training and testing sets by randomly selecting 400 samples from the collected dataset, as described in Table 4.

**Figure 7:**
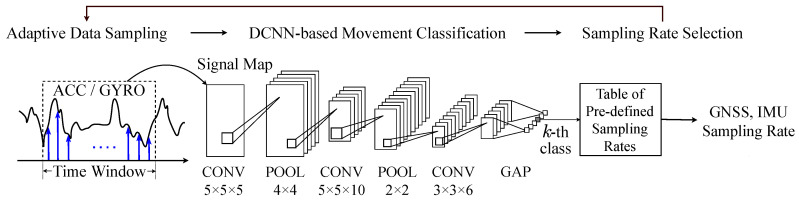
Processing sequence of the proposed DCNN-based sensor control strategy.

Figure 7 depicts the processing sequence of the proposed firmware-level EPTS sensor control that includes the low-cost CNN architecture for on-device processing. Similar to the prior work [43], in every time window, we first gather measurement data from the multi-channel IMU sensor shown in Figure 2 and then make a signal image by rearranging the sensor outputs in the 2D domain. More precisely, the signal image allows making all the sensor channels appear adjacent to each other. Then, as illustrated in Figure 7, we deploy three convolution layers (CONVs) and two max pooling layers (POOLs) to find the temporal features for characterizing the target football activities. For the classifier, we simply introduce an global average pooling layer (GAP) rather than utilizing the computation-intensive fully-connected layers [43], which can provide enough recognition accuracy to be used for categorizing the football activities. Targeting the football activity datasets from the real experimental environments, as a result, the baseline CNN architecture in this work achieves a recognition accuracy of 98.29% while requiring 7.56 KB for storing a whole network model based on 32 bit floating-point numbers.

To make the lightweight processing suitable for the on-device DCNN processing, the proposed DCNN architecture is further optimized by applying the quantization method [48] to represent each network parameter with an 8 bit fixed-point number. In addition, the layer-fusing method from [49] is utilized to merge two adjacent processing layers: one convolution layer and the following pooling layer, making a single processing layer associated with fewer parameters. Table 5 compares the proposed DCNN architecture with the previous method from [43], which provides the smallest model size among existing works, as summarized in Table 3. For fair comparisons, we newly trained the prior work [43] by using the custom dataset of football activities shown in Table 4. Applying the post-training of 8 bit fixed-point parameters, note that we can remarkably compress the model size by 76.3% without degrading the algorithm-level performance. By only using the simple integer-based operations, note that the proposed quantized network even reduces the computing time for recognizing the football activities by 58.3% using the commercialized MCU module when compared to the previous state-of-the-art work using floating-point numbers. It is also possible to reduce the complexity of the prior design by exploiting the fixed-point number system. As depicted in Table 5, however, the aggressive quantization severely degrades the algorithm-level performance of the DCNN model in [43] as it necessitates intensive accumulations for realizing the fully-connected layer. Therefore, the proposed DCNN solution adopting cost-aware optimization schemes is a suitable option to recognize football activities at the resource-limited EPTS device.

### 3.3. DCNN-Based Sensing Rate Control for Energy-Optimized EPTS Operations

After categorizing the current football activity with the proposed lightweight DCNN architecture accepting the IMU measurements, it is possible to adjust the sampling frequency of the GNSS module for reducing the overall energy consumption of the EPTS device, which is the most energy-consuming component, as described in Table 1. Due to the accurate classification results shown in Table 5, it is easy to expect that the proposed activity-based control successfully maintains the amount of position errors with fewer GNSS samples when compared to the baseline EPTS operations shown in Figure 4b. Compared to the straight-forward GNSS control that exploits the speed information only, the proposed technique obviously offers a better control option that further reduces the number of redundant GNSS samples, consequently relaxing the overheads to activate the power-hungry satellite accessing.

It is also possible to reduce the number of sensing operations on the IMU unit, which are used to make the signal image for CNN-based football activity recognition. Therefore, it is required to consider two sensing rates of the GNSS and IMU modules at the same time to find the optimal configuration in terms of energy consumption. For the sake of simplicity, we define $f_{G}$ and $f_{I}$ to denote the sampling rates of the GNSS and IMU units, respectively, which are used for developing the practical algorithm for developing the energy-optimized EPTS operations. Considering the allowable rates for each sensing device, the candidate set *S* is defined by including the possible pairs of $f_{G}$ and $f_{I}$, where $f_{G}\in \left\{1\;\mathrm{Hz},2\;\mathrm{Hz},4\;\mathrm{Hz},10\;\mathrm{Hz}\right\}$ and $f_{I}\in \left\{10\;\mathrm{Hz},20\;\mathrm{Hz},50\;\mathrm{Hz},100\;\mathrm{Hz}\right\}$. For each football activity, we can find the optimal sensing-rate pair $\left({\hat{f}}_{G},{\hat{f}}_{I}\right)$, which can be obtained by solving the following problem:

(2) $$\left({\hat{f}}_{G},{\hat{f}}_{I}\right)=\underset{(f_{G},f_{I})\in S}{arg min}G\left(f_{G},f_{I}\right)$$

Note that the cost function G(·) is newly introduced as follows:

(3) $$G\left(f_{G},f_{I}\right)=\alpha \times P\left(f_{G},f_{I}\right)+\left(1-\alpha \right)\times E\left(f_{G},f_{I}\right),$$

In the proposed cost function, $P(f_{G},f_{I})$ and $E(f_{G},f_{I})$ reflect the power consumption and the sensing error, respectively, where the hyper parameter α provides the scaling factor between two metrics. More specifically, $P(f_{G},f_{I})$ is simply calculated as:

(4) $$P\left(f_{G},f_{I}\right)=P_{G}\left(f_{G}\right)+P_{I}\left(f_{I}\right),$$

where $P_{G}\left(f_{G}\right)$ and $P_{I}\left(f_{I}\right)$ denote the power consumption of the GNSS and IMU modules running at the given sampling rates, respectively. On the other hand, the second part of the cost function in Equation (3), i.e., $E(f_{G},f_{I})$, can be formulated as follows:

(5) $$E\left(f_{G},f_{I}\right)=\beta \times E_{d}\left(f_{G},f_{I}\right)+\left(1-\beta \right)\times E_{s}\left(f_{G},f_{I}\right)$$

For the given configuration of the sampling rates, note that $E_{d}\left(\cdot \right)$ and $E_{s}\left(\cdot \right)$ are calculated by using Equation (1), evaluating the errors in the distance and speed, respectively. We also define the second hyper parameter β that can adjust the ratio of the contributions between two errors.

Due to the impractical number of configurations defined by the candidate set *S*, it is impractical to find the optimal set $\left({\hat{f}}_{G},{\hat{f}}_{I}\right)$ by solving Equation (3) directly. For the practical solution, as described in Algorithm 1, we propose an iterative way to find a near-optimal configuration $\left({\tilde{f}}_{G},{\tilde{f}}_{I}\right)$, which still provides the energy-efficient, yet accurate EPTS operation. Starting from the initial configuration $\left(f_{G}^{\left(0\right)},f_{I}^{\left(0\right)}\right)=\left(10\;\mathrm{Hz},100\;\mathrm{Hz}\right)$, for each football activity described in Table 2, we first reduce the sampling rate of the GNSS module, which minimizes the proposed cost function. Then, the IMU module is adjusted to find the better configuration. As described in Algorithm 1, this process is repeated until there is no change for the two sensing rates. Note that we always find the change of $f_{G}$ first, as the energy consumption caused by the GNSS module is much larger that that of the IMU device, as reported in Table 1. Without evaluating the complex cost function in the excessive number of times, as a result, we can simply get all the practical near-optimal configurations of sensing options for the target football activities, which is described in Table 6. Compared to the threshold-based approach shown in Table 2, which is a straight-forward way of using the speed information from the GNSS module directly, note that the proposed scheme provides a very aggressive control strategy with the high-quality DCNN-based activity classification, leading to the energy-optimized EPTS operations.

As we actively reduce the sensing rate of the IMU module, it is necessary to design an alternative way to construct the signal map, especially for the reduced number of IMU samples. In this work, we apply the linear interpolation to fill the IMU sensing data at the disabled time positions. By preserving the size of the input feature maps for different sensing rates, as a result, we can reuse the proposed DCNN architecture trained for the IMU sampling rate of 100 Hz, when the IMU module even goes to the power-saving mode measuring fewer samples. In other words, the proposed pre-processing always generates the same input format to the pre-designed network for the initial configuration of the sampling rates, i.e., $\left(f_{G}^{\left(0\right)},f_{I}^{\left(0\right)}\right)$, reducing the training overheads to consider the different configurations.
**Algorithm 1:** Iterative method for finding the near-optimal sensing-rate configurations.
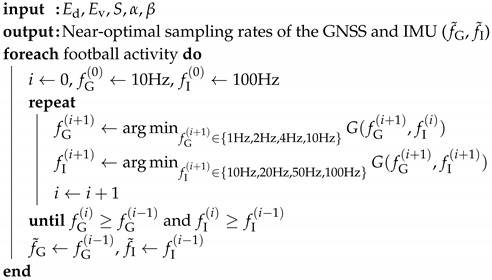


## 4. Experimental Results

**Figure 8:**
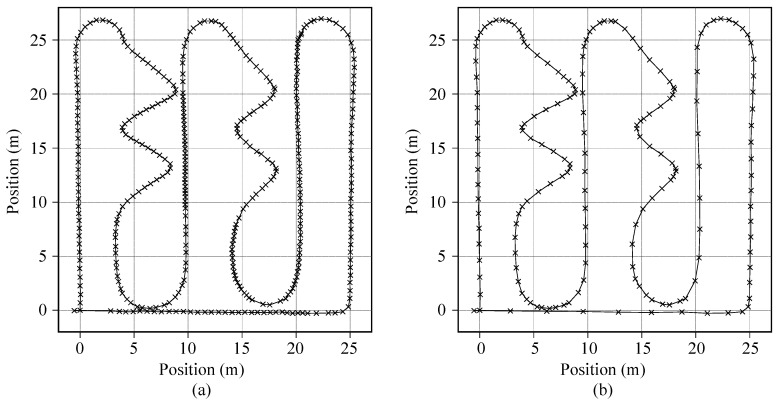
The recovered trajectories derived from (**a**) the straight-forward method and (**b**) the proposed DCNN-based scheme.

To verify the effectiveness of the proposed DCNN-based energy-efficient EPTS operations, we installed several firmware programs at the prototype EPTS device: (1) performing the baseline EPTS operations, (2) reducing the GNSS sampling rate with the straight-forward speed checking, and (3) reducing both $f_{/G}$ and $f_{I}$ by utilizing the proposed lightweight DCNN algorithm. Based on the standardized trajectory test shown in Figure 4a, the recovered paths from the reduced GNSS samples are shown in Figure 8. In both approaches adjusting the sampling rates, we utilized fewer points to yield similar paths when compared to the results of the baseline operations shown in Figure 4b. As we removed only the redundant sensing operations, which is supported by the accurate recognition of football activities with the proposed DCNN architecture, note that the measured trajectory in Figure 8b uses the fewest sampling points compared to the other approaches shown in Figure 4b and Figure 8a.

**Figure 9:**
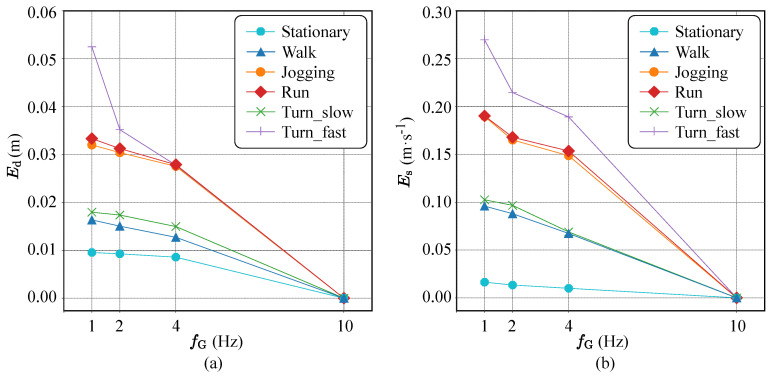
Measurement errors for different football activities in terms of (**a**) distance and (**b**) speed ($f_{I}=100\;\mathrm{Hz}$).

**Figure 10:**
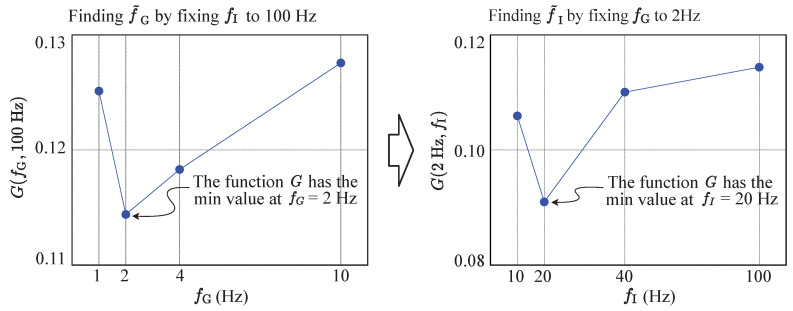
Evaluating the objective function using Algorithm 1 to find the near-optimal sensing-rate configuration of the *turning_slow* activity.

In order to show the quantitative analysis of the proposed DCNN-based EPTS operation, for different sampling rates of the GNSS module, the amount of distance and speed errors is detailed in Figure 9a and Figure 9b, respectively. Note that the optimal $f_{G}$ can be different for each football activity, and the proposed sensor control strategy shown in Table 6 successfully selects the valid option, reducing the measurement errors for the standardized trajectory test. For example, the proposed approach automatically sets $f_{G}=2\;\mathrm{Hz}$ when the CNN operation detects the *turning_slow* activity, which consumes the minimum energy without increasing the distance errors, as shown in Figure 10. On the other hand, the straight-forward method reduces the sampling rates depending only on speed, which requires more samples for turning movements as shown in Figure 8a. Moreover, the proposed DCNN-based control method allows turning off the IMU operations for a while, further reducing the energy consumption of the EPTS device.

Table 7 finally compares the different firmware solutions on the prototype EPTS device in terms of the energy consumption and the sensing errors. Note that the additional firmware overheads to activate the on-device DCNN recognition can be negligible due to the simplified network architecture, increasing the power consumption of the MCU by only less than 1%. Considering the acceptable amount of errors in the practical match scenarios [15,29], as a result, the proposed DCNN-based sensing-rate adjustment saves the power consumption of the GNSS module by 38% when compared to the baseline EPTS operation. Moreover, we can save the IMU power by 34%, whereas the straight-forward way cannot reduce the power consumption of the IMU module. Decreasing the sample rates of the GNSS and IMU modules on demand, as a result, the proposed approach saves the overall power consumption of EPTS operations by 28% and 15% when compared to the baseline system and the straight-forward method, respectively. Note that we still provide attractive data quality, achieving the distance and the speed errors of 0.071 m and 0.657 $m\cdot s^{-1}$, respectively. Therefore, the proposed on-device DCNN processing offers a promising solution for reducing the energy consumption of the EPTS device without degrading the quality of the acquired data.

## 5. Conclusions

In this work, we presented a DCNN-based EPTS device that classifies the movement of football players for reducing the power consumption caused by sensing operations. Compared to the baseline EPTS operations, which fully activate the GNSS and IMU modules, the proposed method dynamically selects the optimal sampling rates of these major sensing components. The novel DCNN architecture is newly introduced for providing the accurate classification of football activities, and several optimization schemes are proposed to enable the on-device DCNN processing with the compressed DCNN structure. Experimental results with the prototype EPTS device reveal that the proposed schemes can drastically reduce the amount of sensing data without degrading the measurement errors, accordingly reducing the power consumption by 28%.

## Figures and Tables

**Table 1:** Power consumption of each component for performing the baseline operation.

	GNSS	IMU	MCU
Current	58.0 mA	22.0 mA	25.0 mA
Power	191.4 mW	72.6 mW	82.5 mW
Proportion	55.2%	21.0%	23.8%

**Table 2:** Football activity types in this work.

Classification	Definition	Speed Range	GNSS Rate (Straight-Forward)
Stationary	Staying in one spot	<0.27 $m\cdot s^{-1}$	1 Hz
Walking	Moving forward by stepping	0.27–1.67 $m\cdot s^{-1}$	2 Hz
Jogging	Moving forward at a slow, monotonous pace	1.67–3.32 $m\cdot s^{-1}$	4 Hz
Running	Moving forward at a high intensity	>3.32 $m\cdot s^{-1}$	10 Hz
Turning_slow	Arc or semicircular motion at a slow pace	–	–
Turning_fast	Arc or semicircular motion at a fast pace	–	–

**Table 3:** Summary of the previous DCNN-based human activity classifiers.

Method	Dataset	Accuracy	Size
Zeng et al. [40]	[46]	96.88%	173.56 KB
Chen and Xue [41]	[41]	93.80%	100.17 KB
Ha et al. [42]	[47]	97.92%	211.40 KB
Jiang and Yin [43]	[45]	97.01%	8.27 KB

**Table 4:** Summary of acquired samples for training the proposed DCNN.

Dataset	Stationary	Walking	Jogging	Running	Turning_slow	Turning_fast
Training set	5028	5288	4988	3716	3548	3744
Testing set	400	400	400	400	400	400

**Table 5:** Implementation results of DCNN operations at the prototype EPTS device.

Model	Accuracy	Latency	Size
Jiang and Yin [43], 32b floating-point	98.13%	115 ms	8.27 KB
Jiang and Yin [43], 8b fixed-point	89.42%	55 ms	2.60 KB
Proposed, 32b floating-point	98.29%	59 ms	7.56 KB
Proposed, 8b fixed-point	98.12%	48 ms	1.96 KB

**Table 6:** Near-optimal sensing-rate configurations from Algorithm 1.

Classification	${\tilde{f}}_{G}$	${\tilde{f}}_{I}$
Stationary	1 Hz	10 Hz
Walking	1 Hz	20 Hz
Jogging	2 Hz	10 Hz
Running	2 Hz	10 Hz
Turning_slow	2 Hz	20 Hz
Turning_fast	4 Hz	10 Hz

**Table 7:** Performance of different firmware solutions.

Scheme	Power Consumption				Measurement Error	
	GNSS	IMU	MCU	Total	$E_{d}$ (m)	$E_{s}$ ($m\cdot s^{-1}$)
Baseline	191.4 mW	72.6 mW	82.5 mW	346.5 mW	0.076	0.679
Straight-forward	137.5 mW	72.6 mW	82.5 mW	292.6 mW	0.073	0.669
Proposed	117.8 mW	48.2 mW	82.5 mW	248.4 mW	0.071	0.657
